# Protection from avian influenza H5N1 virus infection with antibody-impregnated filters

**DOI:** 10.1186/1743-422X-8-54

**Published:** 2011-02-08

**Authors:** Yoichiro Kamiyama, Kazuhide Adachi, Ekowati Handharyani, Retno Damajanti Soejoedono, Takayuki Kusano, Marie Inai, Masaya Tsukamoto, Seizaburo Kashiwagi, Yasuhiro Tsukamoto

**Affiliations:** 1 Life Science Research Laboratories, Research & Development Management Headquarters, Fujifilm Corporation, 577 Ushijima, Kaisei-machi, Ashigarakami-gun, Kanagawa 258-8577, Japan; 2 Department of Animal Hygiene, Graduate School of Biology and Environmental Sciences, Kyoto Prefecture University, 1-5 Nakaragicho, Kyoto 606-8522, Japan; 3 Faculty of Veterinary Medicine, Bogor Agriculture University, JL. Agatis, Kampus IPB Darmaga, Bogor 16680, Indonesia; 4 National Kyushu Medical Center, 1-8-1 Chiyukihama, Chuo-Ku, Fukuoka 810-8563, Japan

## Abstract

There is worldwide concern over the possibility of a new influenza pandemic originating from the highly pathogenic avian H5N1 influenza viruses. We herein demonstrate that functional air filters impregnated with ostrich antibodies against the hemagglutinin of the H5N1 virus protect chickens from death by H5N1 transmission. These results suggest that the use of ostrich antibody-impregnated filters might be a powerful way to prevent the transmission of H5N1.

## Findings

The highly pathogenic avian influenza H5N1 viruses can spread by transmission between domestic and wild birds from Hong Kong, where it was first detected, across Asia, Africa, and into northern Europe [1,2]. Should the H5N1 virus develop the ability to spread efficiently between humans, there would be a high risk of a worldwide pandemic, causing considerable mortality and economic disruption [3-5]. Vaccination is a mainstay of influenza prevention, with annual vaccination recommended for adults and children at a high risk for infection; efforts to prevent person-to-person transmission are also important [3-6]. It has been recommended that health-care facilities implement a universal respiratory hygiene strategy [7,8].

There is an increasing use of antibodies for research, diagnosis, and therapeutic purposes. However, the antibodies from experimental mammals, including the mouse and rabbit, are not well-adapted for industrial usage because of their high production costs. Recently, we have developed a convenient method for the mass-production of antibodies by using ostrich (*Struthio camelus*) eggs [9]. Therefore, it is strongly believed that the ostrich egg may be an excellent antibody source for industrial and medical purposes. Previously, we succeeded in the mass production of ostrich antibodies against the highly pathogenic avian H5N1 influenza virus by immunization of the ostrich layers with viral hemagglutinin (HA). The antibodies have strong neutralization activities against H5N1 infectivities, and the lethality of H5N1 infected birds was dramatically decreased by the direct injection of ostrich antibodies [9]. In the present study, we focused on the application of ostrich antibodies against H5N1 infection. Because the influenza is transmitted by droplet infection [10], air-purification is one of the major factors in preventing influenza viral transmission among individuals. Therefore, we developed a functional air-purification filter coated with anti-influenza antibodies, and examined whether these filters decreased the risk of infection in patients. We herein show that the filters impregnated with ostrich antibodies against HA antigens inhibit the transmission of the H5N1avian influenza virus.

We previously developed a functional air filter impregnated with ostrich antibodies against various influenza viruses, including H5N1 (*Fujifilm Corporation*, *Japan*), and have confirmed that viruses trapped in the filters were effectively inactivated by an antigen-antibody reaction; the infectivities of H5N1 to canine culture cells (MDCK) were drastically inhibited after passing through the antibody filters. In addition, we confirmed that the antibody on a solid surface specifically reacted with a protein antigen supplied from a gas phase under the nominal ambient condition, by using FRET (fluorescence resonance energy transfer) signal as a mean to quantify the reaction between pairs of antibody labeled with a donor fluorophore and antigen labeled with an acceptance fluorophore [11]. In the present study, a convenient model for droplet- or fecal infection of influenza viruses was used. Boxes (12 × 16 × 30 cm) composed of coarse mesh- or antibody-impregnated or untreated filters were set up. Each box has three openings, of which total area is 388 cm^2^, on both flanks and ceiling. The effective amount of the ostrich antibody impregnated in the nonwoven fabric filter coping with H5N1 is ca. 175 μg per the box. Normal white leghorn chicks were housed in these filter covered boxes with food and water. Chicks at 10 days of age were intranasally inoculated with avian influenza virus A/Bogor 2/IPB/H5N1 at a dose of 10^5 ^TCID_50_, and were then housed around the filter covered boxes including non-inoculated chicks. At 6 days post-inoculation, the mortality of chicks in the filter covered boxes was calculated. The survivors were sacrificed with a pentobarbital solution, and the lungs were removed and fixed in buffered formalin for the histopathological and immunohistochemical analyses of viral infection.

**Figure 1:**
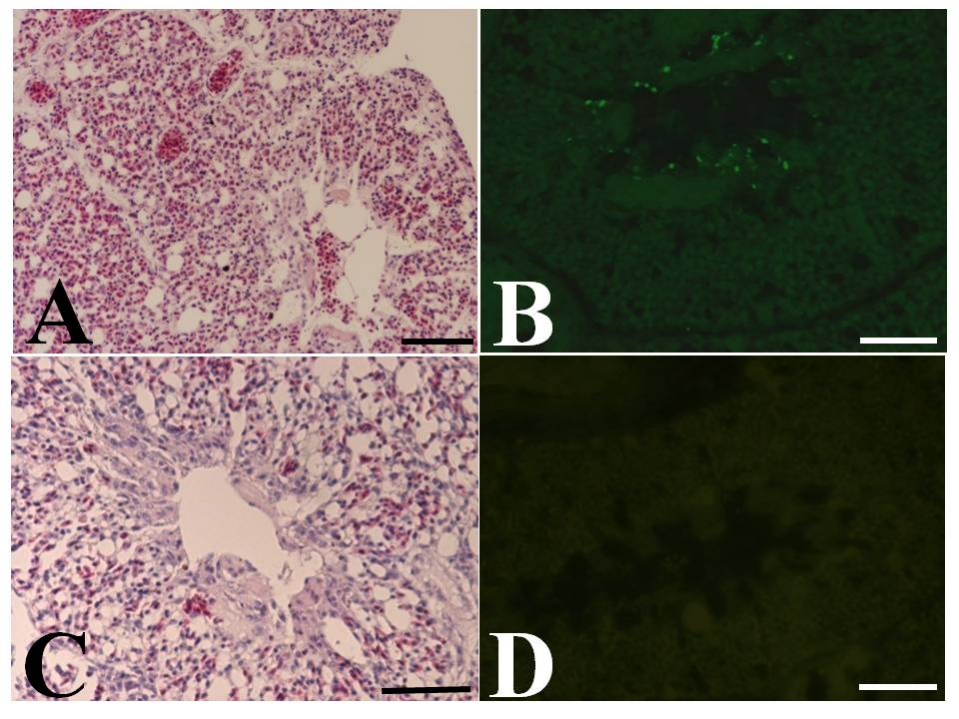
**Histopathology and immunohistochemistry examination of H5N1 antigens**. The survival chicks were sacrificed, and the lungs were removed and fixed in buffered formalin. Hematoxylin and eosin staining were performed on paraffin sections as previously described. For the immunohistochemical analyses, a monoclonal antibody against influenza A was incubated on the sections. A FITC-conjugated secondary antibody was incubated. Severe inflammation (A) and viral antigens (B) were observed in the lung of chicks housed in the untreated-filter box. In contrast, obvious histopathological legions (C) and viral antigens (D) were not detected in the lungs of chicks housed in antibody filter boxes. Scale bars represent *100 μm*.

Most of the surrounding H5N1-inoculated birds died at 3 days post-inoculation. As shown in Table 1 all birds escaped from death when they were housed in antibody-filter covered boxes, whereas the mortality of the birds in coarse mesh- and untreated-filter covered boxes was significantly higher. Histopathology and immunohistochemistry experiments revealed that severe inflammation and viral antigens were present even in the survivors in both coarse mesh- and untreated-filter covered boxes; in contrast, no obvious reactions were present in any chicks that were contained in the antibody filter covered boxes (Figure 1). These findings suggested that the antibody filters rescued the chicks from the viral transmission by H5N1-infected birds. Accordingly, the H5N1 viruses via droplet or fecal infections [12] from infected birds might be neutralized on the filters, because the HA of viruses was masked with ostrich antibodies, and could not enter the host cells; the viral particles from the filter had no infectivity in the animals. The avian influenza virus is highly infectious to the chickens compared to humans because of the distributions of receptor on host cells [13,14]. Due to the fact that the complete inhibition of H5N1 transmission was found in the present chick experiment, similar effects may therefore be observed in human cases. For this reason, antibody filters will likely be a powerful tool for protection against avian influenza transmission.

**Table 1:** Mortality and infection rate

		Number of deaths										Filter covered box		
							
Type of partition	Number of chicks	day 1	day 2	day 3	day 4	day 5	day 6	Toal number of deaths	Mortality	dead or infected	Infection ratio	Number of box	Number of infected box	Infection rate (%)
(a) Mesh filter	24	0	0	0	0	5	18	23	96%	24	100%	6	6	100
(b) Untreated filter	20	0	1*	0	0	0	7	7	37%	15	79%	5	5	100
(c) Antibody filter	20	0	0	0	0	0	0	0	0%	0	0%	5	0	0

Inoculated chicks	48	0	0	42	6	0	0	48	100%	-	-	-	-	-

(a) polypropylene net with 3.7 mm square mesh-, or (b) untreated nonwoven fabric middle efficiency dust filter-, or (c) antibody impregnated same grade filter as (b), were attached to the three sides of the box cages (12 × 16 × 30 cm). Four male chickens weighing 80 g (10 days of age) were housed with food and water in each box cage covered with net or filter. Other chicks were intranasally inoculated with Highly Pathogenic Influenza virus A/Bogor 2/IPB/H5N1(10^5^TCID_50_/bird). Three infected chicks were housed with food and water at the central area, closely surrounded by the filter boxes. Most of infected chicks at the central area died at 3 days post viral inoculation. At day 6, the surviving chicks in filter partitioned boxes were counted, and the mortality was calculated. In the histopathological and immunohistockemical analyses, the lungs of chicks were examined, and the box with at least one infected chick was scored as an infected box. * Since one chick in the untreatd filter box died in accidentally, the mortality and infection rate were calculated without this accidental case.

The basic specification of the nonwoven fabric middle efficiency dust filter used in (b) and (c) is as follows; average thickness: 0.8 mm ± 0.1 mm, average arrestance: ≧70% according to JIS Z 8901 gravitational method, initial pressure loss: 25 Pa @LV 0.5 m/s.

## Competing interests

The authors declare that they have no competing interests.

## Authors' contributions

YT designed research; KA, EH, RDS, TK, MI, MT performed research; YK, YT and SK analyzed data and wrote the paper. All authors read and approved the final manuscript.
